# Reporting of interventional clinical trial results in an academic center: a survey of completed studies

**DOI:** 10.1186/s12874-024-02221-6

**Published:** 2024-04-22

**Authors:** Anne Sophie Alix-Doucet, Constant Vinatier, Loïc Fin, Hervé Léna, Hélène Rangé, Clara Locher, Florian Naudet

**Affiliations:** 1 Research and Innovation Department, CHU Rennes, Rennes, France; 2 Univ Rennes, CHU Rennes, Inserm, EHESP, Irset (Institut de Recherche en Santé Environnement Et Travail)-UMR_S 1085, CIC 1414 [(Centre d’investigation clinique de Rennes)], F- 35000, Rennes, France; 3 Centre Hospitalier Universitaire, Hôpital de Pontchaillou, INSERM U 1242, Université Rennes 1, Rennes, France; 4 CIC 1414 [(Centre d’Investigation Clinique de Rennes)], Univ Rennes, CHU Rennes, Inserm, Institut Numecan (Nutrition, Métabolismes Et Cancer) -UMR_S 1317, Rennes, France; 5 Institut Universitaire de France (IUF), Paris, France

**Keywords:** Open Science, Clinical studies, Results, Publication, Audit

## Abstract

**Background:**

The dissemination of clinical trial results is an important scientific and ethical endeavour. This survey of completed interventional studies in a French academic center describes their reporting status.

**Methods:**

We explored all interventional studies sponsored by Rennes University Hospital identified on the French Open Science Monitor which tracks trials registered on EUCTR or clinicaltrials.gov, and provides an automatic assessment of the reporting of results. For each study, we ascertained the actual reporting of results using systematic searches on the hospital internal database, bibliographic databases (Google Scholar, PubMed), and by contacting all principal investigators (PIs). We describe several features (including total budget and numbers of trial participants) of the studies that did not report any results.

**Results:**

The French Open Science Monitor identified 93 interventional studies, among which 10 (11%) reported results. In contrast, our survey identified 36 studies (39%) reporting primary analysis results and an additional 18 (19%) reporting results for secondary analyses (without results for their primary analysis). The overall budget for studies that did not report any results was estimated to be €5,051,253 for a total of 6,735 trial participants. The most frequent reasons for the absence of results reported by PIs were lack of time for 18 (42%), and logistic difficulties (e.g. delay in obtaining results or another blocking factor) for 12 (28%). An association was found between non-publication and negative results (adjusted Odds Ratio = 4.70, 95% Confidence Interval [1.67;14.11]).

**Conclusions:**

Even allowing for the fact that automatic searches underestimate the number of studies with published results, the level of reporting was disappointingly low. This amounts to a waste of trial participants' implication and money. Corrective actions are needed.

**Trial registration:**

https://osf.io/q5hcs

**Supplementary Information:**

The online version contains supplementary material available at 10.1186/s12874-024-02221-6.

## Introduction

The latest version of the Declaration of Helsinki from the 64th WMA General Assembly, Fortaleza Brazil October 2013 [1], includes a section on “*Research Registration and Publication and Dissemination of Results*" where it is pointed out that results must be disseminated regardless of the direction or strength of the study findings. Indeed, it is stated that “*negative and inconclusive as well as positive results must be published or otherwise made publicly available*”. Two years later, in 2015, the World Health Organization (WHO) published a new statement on the public disclosure of clinical trial results [2] where they also reaffirmed that “*there is an ethical imperative to report the results of all clinical trials*”. In the USA, a requirement of this nature for drugs, biologics, and medical devices subject to Food and Drug Administration (FDA) regulation was established in 2007 [3]. Other countries and funders have since implemented similar policies [4]. Since 2018, a similar requirement has been implemented in Europe for clinical trials on medicinal products for human use [5, 6].

The proportion of clinical trials reporting their results in timely manner can be monitored with tools such as the FDAAA Trials Tracker [7] for trials initiated in the USA, or the EU Trials Tracker [8] for trials initiated in the European Union. Although there has been improvement in reporting of results in the EUCTR, with an increase from 50% in 2018 to 84% in late 2022 [9], communication of results either by posting results on registries or by publications remains suboptimal [10, 11]. The lowest proportions are observed for academic sponsors [12], with some heterogeneity across countries, with France registering among the lowest proportions [13]. In addition, the scope of EUCTR surveillance is limited to clinical trials on drugs, and therefore does not include most trials sponsored by academic centers. For instance drug trials only accounted for 18% of interventional trials conducted in France in 2022 [14]. The French Open Science Monitor [15] is an initiative of the French Ministry of Higher Education, Research and Innovation with broader scope, since it is not restricted to drugs. It tracks open science features for both interventional and observational studies [16]. It uses both "EUCTR" and "ClinicalTrials.gov" [17].

Since there is agreement on the fact that the reporting of clinical trial results should be considered as a strategic parameter to monitor in biomedical research institutions [18], we carried out a survey of all completed studies in our academic center (Rennes University Hospital, France), exploring the dissemination of its interventional clinical study results. Interventional studies were defined according to clinicaltrials.gov definition [19]. We aimed to describe the status of interventional studies from the point of view of the reporting of results. More specifically, we explored 1/ how far completed studies had made their results available (we compared the trial status based on the French Open Science Monitor results with the actual status of the trial as assessed by systematic searches) 2/ the features associated with non-report of data and 3/ the feasibility of interventions to improve the level of reporting of clinical trial results.

## Methods

The methods of this cross-sectional study were pre-specified and documented in a protocol registered on the Open Science Framework on January 13, 2023 [20].

### Eligibility criteria

We surveyed completed interventional studies identified via the French Open Science Monitor [15].

### Search strategy and study selection

We obtained an extraction of all interventional studies tracked by the French Open Science Monitor on December 9, 2022 by contacting the team in charge of developing the Monitor. As all studies were recorded on the "ClinicalTrials.gov" registry, we used the National Clinical Trial (NCT) number as the single reference. The Monitor automatically records results that are either "posted" or identified as "published" on the registries. "Posted" results correspond to the posting of summary results of a study in at least 1 of the 2 registers available (EUCTR or clinicaltrials.gov). "Published" results correspond to scientific publications with a Digital Object Identifier (DOI) and with at least one author with a French affiliation [16]. The studies retrieved by the French Open Science Monitor (BSO) and used in this audit correspond to those with a completion date between 2011 and 2021.

### Systematic searches for reporting status

For each study, we used the following strategy to ascertain reporting status. First, we retained the reporting status identified by the French Open Science Monitor. Then we chained this extraction with the internal database of Rennes University Hospital using Easydore® software, which references all studies carried out there, with their administrative and budgetary data as well as any associated published results. For each publication identified, one researcher (ASAD) recorded whether it was a primary or secondary publication. A primary publication includes the results for the primary endpoint (as registered in "ClinialTrials.gov or EUCTR") for all patients included in the study. Secondary publications encompass other publications (ancillary or other: review, methodology, etc.) that do not report the primary outcome for the whole trial population. If the reviewer was not able to make this distinction, a second researcher (CL or FN) was consulted. In the most difficult cases, the 3 researchers contacted the investigators before deciding whether the publication should be considered as primary or secondary. If no primary publication was identified at this stage, we searched first on Google Scholar using the study NCT number. Then, if no primary publication was found, an e-mail was sent to the principal investigator (and associated investigators in the absence of a response). We sent two reminders at intervals of 3 days if there was no response. If no primary publication had been identified in the previous steps, one researcher (ASAD) searched for articles on Pubmed using the name of the principal investigator and a publication date later than the study completion date. If an article was identified in this way, a second reviewer was consulted before including it (FN, CL).

### Survey of principal authors

We sent 2 short e-mail surveys (see WebAppendix) to authors (PIs and/or associate investigators) of studies identified as having no primary publication, in order to ascertain the reason(s). They were also asked whether they planned a primary publication for these studies. When the answer was positive, they were asked to specify the stage reached in the publication process (analysis/writing/submission/evaluation). A final question aimed to identify the reasons for the absence of a primary publication.

### Outcomes

Our primary outcome was the proportion of primary results available for studies completed (date of the last patient visit) more than one year before the start of the present survey. This time frame aligns with the requirements of the clinical trial directive 2001/20/CE [21] for drug trials and is consistent with WHO best practices [22].Our secondary endpoints were 1/ time from study completion to the reporting of results, 2/ discrepancies between results of our systematic searches and the results identified by the French Open Science Monitor, 3/ total budget for studies with no reported results, 4/ total expected budget that would be received after communication of their results (for the studies funded by the French Direction Générale de l'Offre de Soins (DGOS), where 10% of the budget is released after results are reported), 5/ reasons provided for studies with non-reported results. All these outcomes were extracted by one researcher (ASAD) either automatically when feasible or manually. In case of doubt, arbitration took place with CL or FN.

### Pilot test of an intervention

In case of discrepancy between the French Open Science Monitor and the actual status of a particular study identified by our searches, we updated registries (clinicaltrials.gov) by adding the link to the corresponding publication(s). LF and ASAD are entitled to make changes to trial registry records for studies as employees of the study sponsor, the “Direction de la recherche et de l’innovation” at Rennes University hospital. We recorded the time required to complete this task. In addition, study results were collected on 3 purposively selected studies (selected for their differing designs) and the synthesis of their results was posted on the ClinicalTrials.gov or EUCTR databases. We noted the time spent on these actions.

### Statistical analysis of results

The statistical analysis was carried out using R studio software (version 4.2.2 (2022–10-31)) by CV. The main analysis was descriptive. It consisted in presenting numbers (and percentages) for categorical variables and median (and inter quartile range, IQR) for quantitative variables. For discrepancies with the French Open Science Monitor, we computed the diagnostic accuracy of the Monitor in identifying studies actually published (i.e. we computed sensitivity, specificity, positive and negative predictive values). An exploratory analysis investigated associations between the following variables and the reporting of results: 1/ time since study completion (between last visit of the last patient and completion date), 2/ trial sample size (number of participants recruited), 3/ total budget, 4/ study design (randomized/non-randomized), 5/ study outcome for primary endpoint (positive/negative), a positive result being defined as a statistically significant result (in either direction).This analysis used a logistic regression (first in a univariate analysis and then in a multivariate analysis). Data and codes for these analyses are available on the Open Science Framework [23].

### Change to the initial protocol

For the systematic searches, we slightly revised the order of the different steps initially planned in our protocol in order to make the process more efficient. In addition, we made a distinction between primary and secondary publications, since we identified several studies with secondary publications and no primary publication of the results. Our initial protocol did not explicitly mention an exploration of the diagnostic accuracy of the French Open Science Monitor, so it was added a posteriori because we found this information interesting and important. As part of our survey of the study authors, we asked an additional question to find out whether investigators in unpublished studies were still planning to publish results, and if so the stage in this process.

## Results

**Fig. 1:**
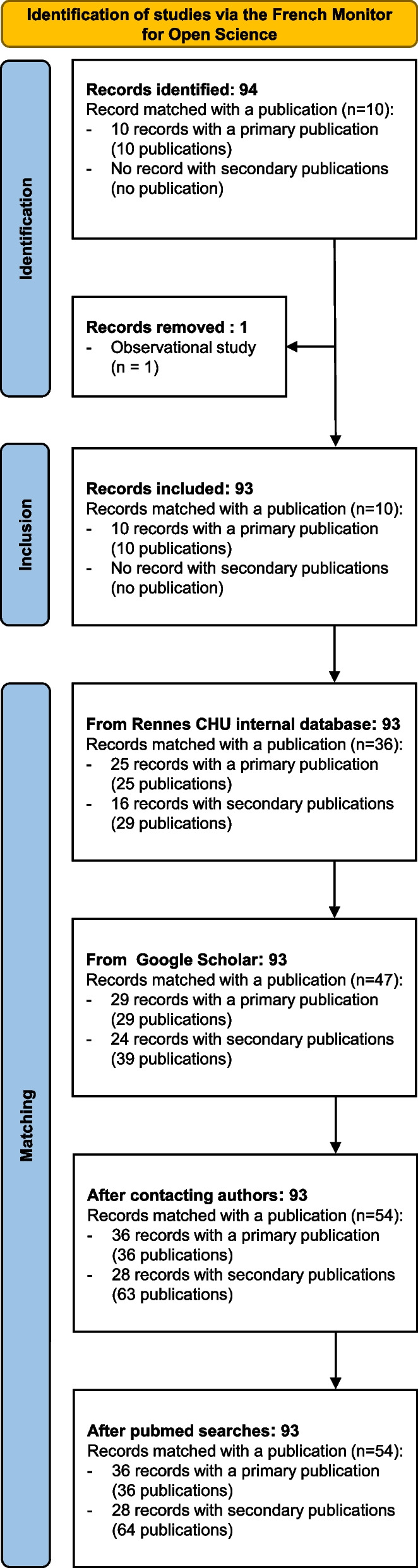
Study flow-chart

A total of 94 eligible trials were retrieved from the French Open Science Monitor. Of these, 1 was excluded because it was a non-interventional study and 93 interventional trials were included in the survey. For 10/93 (11%) the French Open Science Monitor automatically indexed trial publications (all were primary publications). None of these studies had results posted in either of the 2 registries (ClinicalTrials.org nor EUCTR). Details of our searches for published results are provided in Fig. 1. Table 1 presents a detailed description of these studies.

**Table 1:** Description of all studies included

	**All studies (93)**	**Randomized (29)**	**Non-randomized (64)**
**Study type** *(n (%))*			
Drug	18 (19%)	9 (31%)	9 (14%)
Biological	12 (13%)	0 (0%)	12 (19%)
Behavioral	4 (4%)	1 (3%)	3 (5%)
Device	20 (22%)	8 (28%)	12 (19%)
Diagnostic test	6 (7%)	1 (3%)	5 (8%)
Dietary supplement	3 (3%)	2 (7%)	1 (2%)
Procedure	22 (24%)	7 (24%)	15 (23%)
Other	8 (9%)	1 (3%)	7 (11%)
**Research Domain** *(n (%))*			
Anesthesia, resuscitation	12 (13%)	4 (6%)	8 (28%)
Biology and physiology	6 (6%)	4 (6%)	2 (7%)
Clinical Investigation	3 (3%)	1 (2%)	2 (7%)
Clinical Pharmacology	2 (2%)	2 (3%)	0 0%)
Emergency Departments	2 (2%)	2 (3%)	0 (0%)
Medical Specialties	30 (32%)	23 (36%)	7 (25%)
Neurology	6 (6%)	4 (6%)	2 (7%)
Odontology	1 (1%)	1 (2%)	0 (0%)
Pediatrics	5 (5%)	4 (6%)	1 (3%)
Pneumonology	3 (3%)	1 (2%)	2 (7%)
Psychiatry	4 (4%)	1 (2%)	3 (10%)
Public health	4 (4%)	4 (6%)	0 (0%)
Radiology	6 (6%)	6 (9%)	0 (0%)
Surgery	9 (10%)	7 (11%)	2 (7%)
**Completion year** *(median [IQR])*	2018 [2015; 2019]	2018 [2015; 2019]	2017 [2015; 2019]
**Trial sample size** *(median [IQR])*	50 [25; 120]	80 [33; 140]	49 [25; 114]
**Total budget (Euros)** *median [IQR]*	38834 [19591; 95260]	88131 [37349; 331250]	34805 [11043; 57363]
**Study outcome** *(n (%))*			
Positive	50 (54%)	12 (41%)	38 (59%)
Negative	30 (32%)	13 (45%)	17 (27%)
Unknown	13 (14%)	4 (14%)	9 (14%)

### Primary outcome

**Fig. 2:**
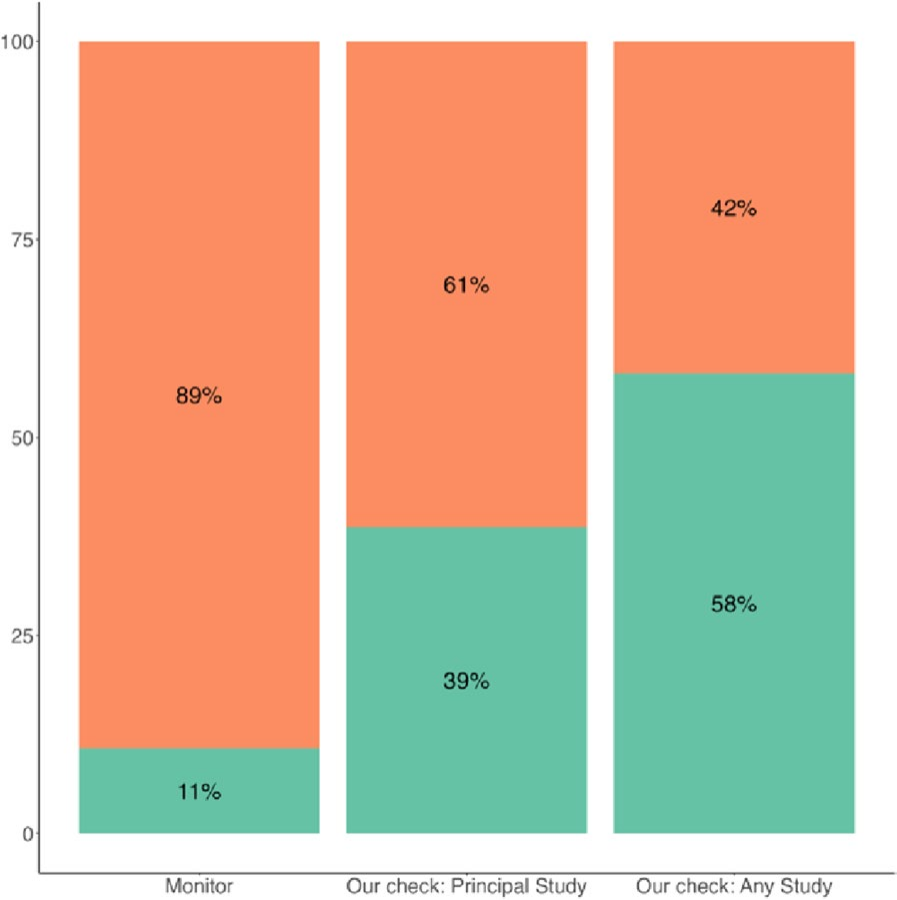
Percentages of published (green) /unpublished (orange) research identified with the Monitor and after our internal checks (primary publications and all publications)

We identified 36/93 (39%) studies with results reported in primary publications (Fig. 2).

In addition, we identified a total of 64 non-primary publications relating to 28 trials. Alongside, some trials had several non-primary publications (with a maximum of 9 non-primary publications for a single study). Among the 28 trials with secondary publications, only 10 also had main results reported in a primary publication.

### Secondary outcomes

For the 36 studies with a primary publication, the average time between completion and publication was 3.0 (IQR 1.96;3.49) years. Non-primary publications were published faster, 0.7 (IQR -1.18; 1.83) years (min = -3.6, max = 5.3) as several of them were actually published before the last visit of the last patient (i.e. they were preliminary results).

Regarding the diagnostic accuracy of the French Open Science Monitor, all studies identified as having results by the Monitor were indeed true positives (specificity = 1; positive predictive value = 1). The Monitor yielded some false negatives, since it did not retrieve 26 of the 36 studies with primary publications (sensitivity = 0.28; negative predictive value = 0.31).

The overall budget for all the studies without available results was estimated to be €5,051,253 for a total of 6,735 trial participants. Six studies without available results were funded by the French DGOS for a total budget of €2,610,481, with €261,047 to be issued by the funder after publication. For five of these 6 studies the investigators questioned indicated publication was "ongoing": two at the analysis stage, one at the drafting stage, and 2 submitted and/or under assessment.

We surveyed the investigators/project managers of 57 studies without an available primary publication: Despite reminders, we did not receive any response for 6 studies. Among the 51 remaining, the primary publication was currently in progress for 27 trials: 8 were in the pre-analysis or analysis stage, 9 were at drafting stage, 8 in the process of being submitted and 2 were being evaluated by journals. For 24 trials, the investigators replied that there were no plans to publish the main results.

**Fig. 3:**
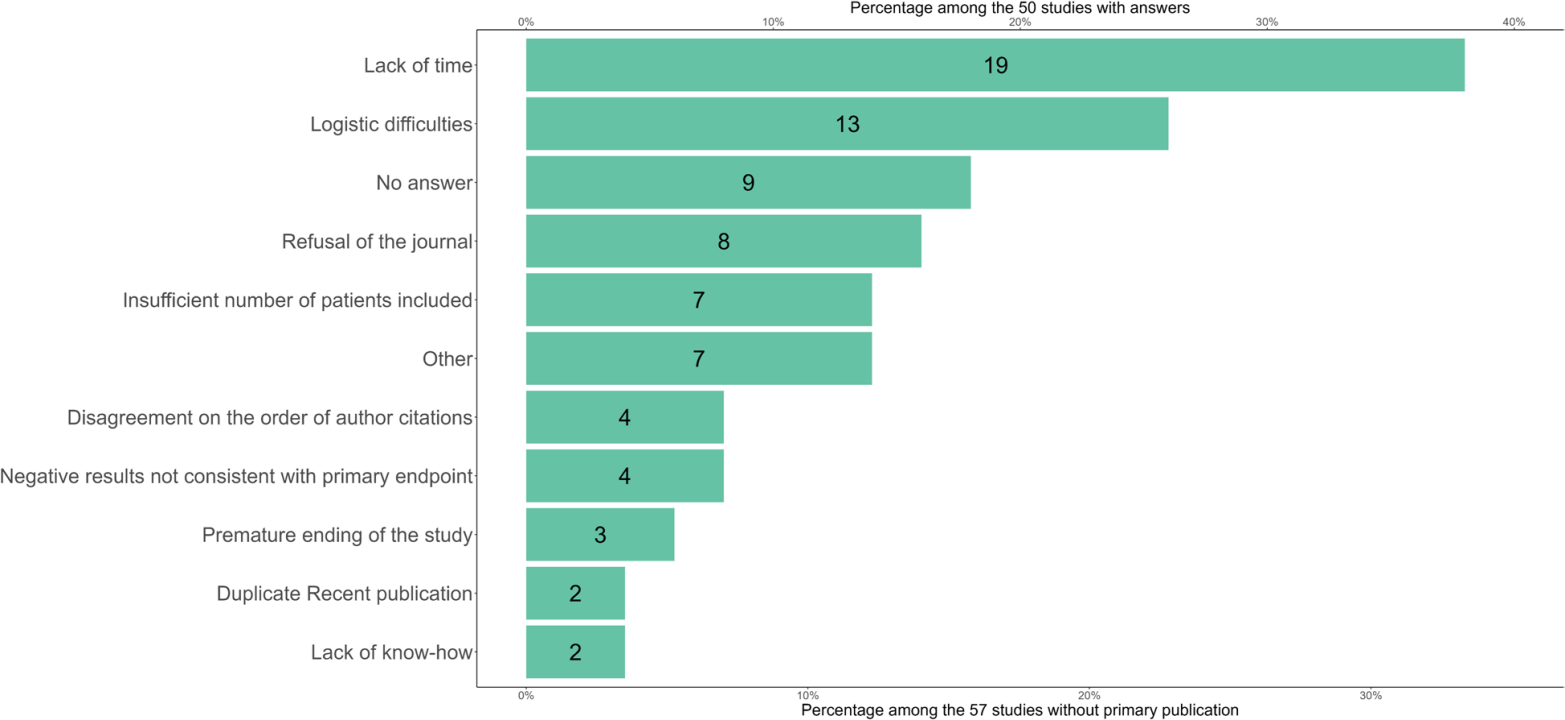
Distribution of the number of answers (green: yes) given for each reason ticked by investigators concerning their studies with non-disseminated results. Percentages are calculated for studies with a response (*n* = 50), and the second set of percentages are for studies without primary publications (*n* = 57)

Among the 57 studies without a primary publication, the investigators/project managers answered for 50 of these studies about the reasons for not publishing any primary results. The two most frequent reasons were lack of time for 18 (32%), and logistic difficulties (i.e. delay in obtaining results or another blocking factor) for 12 (21%). Other reasons are presented in Fig. 3. None of the investigators interviewed stated that publication costs were a reason.

### Exploratory analysis

Table 2 presents the results of the logistic regression exploring factors associated with availability of results. This analysis identified an association between positive results on the primary outcome and the publication of results. None of the other variables explored was associated with publication of results.

**Table 2:** Exploratory analysis performed on all published studies and for variables identified as potentially associated with publication (logistic regression)

Variables	Univariate analysis	Multivariate analysis
	**aOR [CI 95%]**	**aOR (CI 95%)**
**Sample size**	1.00 [0.99; 1.00]	1.00 [0.99; 1.00]
**Design (randomized trials)**	0.91 [0.37; 2.27]	1.31 [0.42; 4.33]
**Study results on the primary outcome (ref = negative):**		
*Unknown*	0.22 [0.03; 1.03]	0.26 [0.04; 1.23]
*Positive*	4.36 [1.65; 12.12]	4.70 [1.67; 14.11]
**Total budget**	0.99 [0.99; 1.00]	0.99 [0.99; 1.00]
**Time since study completion**	1.07 [0.91; 1.26]	1.08 [0.89;1.31]

### Pilot test on an intervention

The ClinicalTrials.gov register was updated for each of the 54 studies with one or more publications (primary and/or secondary with a specific distinction between the two. An overall time of 139.5 min, i.e. around 2h20 was needed to complete the register for all studies. The mean time per study was thus 1min30s (1min30s; 3min22s).

The results available for 2 studies (NCT0197347 and NCT00598026) and for one study (NCT02650609) are being posted into the ClinicalTrials.gov register and Eudract respectively. None could be posted because formatting issues regarding pharmacovigilance data requiring further details than the details provided in the study documents. We estimated that 1 day would be sufficient to post on clinicaltrials.gov and around 5 days on Eudract.

## Discussion

### Statement of principal findings

This cross-sectional study quantified the availability of results for all interventional studies conducted with Rennes University Hospital as an academic sponsor. The proportion of studies with available results was low, especially for primary study results. Automatic monitoring using the French Open Science Monitor identified a proportion of 11%, underestimating the actual percentage (39%). The diagnostic accuracy of the French Monitor for Open Science was indeed imperfect, as it relies on an automatic detection of published results. Although NCT citation is a basic reporting feature that is required by CONSORT [24], it was lacking in the many false negative studies we identified. Even our search based on Google Scholar failed to identify many of the trials, since the registration numbers were not provided even in the full text of the corresponding papers. Therefore, the results of automatic tools could be inaccurate markers of the actual status of reporting of results, with an underestimation of published results. However, the time required to update the status of all published studies in ClincalTrials.gov was minimal, with little added administrative burden for the sponsor. In other words, automatic monitoring can be accurate and very useful only when registries are updated by the sponsor. For studies with published results, the time between completion of a study and its primary publication was much longer (3.0 (IQR 1.96;3.49) years) than the regulatory timeframe (1 year). With 57 out of 93 (61%) studies without available results, the waste of money for the community as a result of non-publication of study results was 5,051,253 euros in our academic center, and accounted for 6,735 trial participants who agreed to take some risks without any known benefit, whether for the scientific community or for the patients.

Investigators failing to publish the primary results of their studies mostly declared that it was a result of lack of time and/or logistic difficulties. This result is in line with a previous survey in France highlighting similar difficulties [25]. A minority (3/61; 5%) declared that non-publication was a result of negative results. Our results suggest that negative results were associated with non-publication, probably indicating the existence of a degree of publication bias, with the selective publication of positive findings as described previously [26].

In 7/61 (11%) instances, the investigators stated that non-publication was a result of manuscript rejection by journals. None of these studies had however posted results on registries, which would indeed be an interesting option in these cases [27].

### Strength and limitations

We were able to collect highly detailed information with a granularity that was difficult to achieve in previous surveys using automatic tools [10] since automatic extractions have some shortcomings, especially in relation to false negative results. In addition, we were able to estimate the number of participants included as well as the wasted cost generated by non-publication. Quantification of research wastage can be a very convincing argument for stakeholders to act.

There are however a number of limitations to our survey. Although we tried to be as exhaustive as possible, despite reminders some principal investigators did not reply to our emails. In some cases, it was difficult to determine whether published results were actually primary or secondary publications. Concerning our survey, certain questions may have been misunderstood by the investigator, potentially introducing the possibility of misclassification bias (e.g. regarding “logistic difficulties” we did not distinguish between “logistic difficulties” before vs. after study completion). Because any judgement is subjective, efforts were made to cross-validate data extraction and we think that we were able to limit the misclassification bias. In addition, our survey reports on the existence of results for a given trial, but we made no qualitative judgement about the accuracy of these results, for instance regarding the existence of spin [28] or other potential bias in the different studies. Moreover, as our study does not involve a random selection of papers, we have to recognize that uncertainty is greater than that reflected by 95% confidence intervals. Finally, these results observed in one specific French academic centre may not be suitable for generalisation to other contexts.

### Perspectives

This survey is a first step in improving transparency for interventional trials at Rennes University Hospital. It enabled us to identify the extent of the problem. One cannot be satisfied with the current situation, which is surely not restricted to this academic centre and is a general problem in France [13]. We identified certain solutions which will be proposed to the research directorate of Rennes University hospital (Table 3). Some simple actions should be considered promptly: the systematic indication of the registration number in abstracts of future publications would largely facilitate automatic indexing and avoid false negatives in the French Open Science Monitor. The sponsor could check this at the time of publication, since authors often request funding for publication costs. We also plan to educate investigators on the importance of open science practices, including a reminder of the value of publishing studies with negative results. In addition, regular reminders could be implemented, every 3 months for example, to investigators with long-standing unpublished studies. Reminders of this type have been proven to work for completed trials that did not comply with the FDA requirements [29]. The regular updating of the ClinicalTrials.gov registry should also be planned or carried out in line with publications sent in by Principal Investigators. Continuous monitoring of transparency indicators could help improve the situation over the long term. Still, this is likely to be insufficient given the extent of the problem.

**Table 3:** Summary of proposals for action

Sponsors
**Exploring and addressing the causes of logistic difficulties**
**Uploading of summary results in the 2 registers**
**Regular study updates on ClinicalTrials.gov**
Pubmed link(s) to publication(s) provided by investigator(s)
**Hiring dedicated staff for the purpose of dissemination of results**
Biometrics, medical writer, SO referent
Dedicate time to restoring unpublished trials
**Extending the policy to observational studies**
**Extending the policy to other open science features (e.g. data-sharing)**
**Implementation of Open Science Indicators to monitor the output of the centre**
**Actions toward investigators**
**Capacity building:**
Open Science practices
Publication of negative results
Systematic inclusion of NCT in abstracts
Systematic reminders for overdue studies
Systematic reminders of regulatory obligations concerning publication of results
**Implementing incentives for publishing results**
Restricting access to initiation of a new study, in case of non-publication of a previous study

Systematic posting of the summary of results in one of the 2 registries (i.e. EUCTR for clinical studies on medicinal products or ClinicalTrials.gov for any other research objects) should be implemented, either by designating a person with a percentage of dedicated work time, or by enabling project managers to be granted time for this specific purpose. This implies dedicating specific resources to open science practices of this nature. Given the skills needed to master these platforms, these resources must be permanent. The EUCTR register should be prioritized, as it has become a regulatory requirement to contribute to it since January 31, 2022 (implementation of European Regulation n°536/2014). It is also very important to have a more precise understanding of the logistic difficulties reported by some of the investigators questioned. Regarding publications, drastic solutions could include setting up a team to restore unpublished trials, in line with the RIAT initiative [30]. The sponsor may also want set up a committee to decide whether or not the investigators of unpublished studies should be allowed to initiate a new study, or should wait until the results of their studies are published and/or posted. This could thus provide appropriate incentive, insofar as the obstacles faced by investigators can all be overcome by appropriate institutional support. Investigators could have the benefit of specific editorial assistance providing appropriate rules for authorship, and with dedicated staff identified as open science referent(s). Lastly, while it is clear that providing access to aggregated data is of course a minimum requirement that is not met at present, the obvious next step will be to provide access to individual patient data. These transformations are necessary to draw on the full potential of medical research [31].

## Conclusion

This survey has shown that the proportion of studies sponsored by our institution that are actually published remains clearly unsatisfactory, even if it is slightly higher than the proportion estimated by the national monitoring system. It is a first step in improving the availability of interventional study results sponsored by Rennes University Hospital. This highlights the extent of the work that is needed to reach full transparency.

### Supplementary Information

**Supplementary Material 1.**

## Data Availability

The datasets generated and/or analysed during the current study are available in the Open Science Framework repository https://osf.io/ag7f2/.
