# Supplementary material for: Reporting of interventional clinical trial results in an academic center: a survey of completed studies
Source: BMC Med Res Methodol. 2024 Apr 22;24:93. doi: 10.1186/s12874-024-02221-6 (PMC11034140; doi:10.1186/s12874-024-02221-6)
Supplement: Supplementary file 1 — Supplementary Material 1. [file 12874_2024_2221_MOESM1_ESM.docx]

**Web Appendix: Two E-mail Surveys**

**First e-mail survey to Investigators (PIs and/or associate investigators) to identify primary publications**

Pr XXX,

At the beginning of the year 2023, the Research and Innovation Direction set out to initiate a census of publications derived from studies promoted by Rennes University Hospital.

The objective is to enhance the Open Science Barometer results ([**https://barometredelascienceouverte.esr.gouv.fr/sante/essais-cliniques/general**](https://barometredelascienceouverte.esr.gouv.fr/sante/essais-cliniques/general)**)**

In this setting, your publication entitled: [Title of the study]

DOI : xxx

was identified in relation to the present study known under the acronym XXX  and NCT XXX

Please could you provide the following information:

The primary objective of your study is: [Primary study outcome]

The number of patients included in this study is: [n]

**Can you confirm that the publication cited above is the primary publication?**

Indeed, we are unable to identify [the number of patients included / the primary outcome]

Do not hesitate to send me other publications linked to this study.

**Otherwise, please confirm that there is no primary publication for this study (including a primary outcome and a completed analysis of all patients included)**

I am at your disposal to discuss this and thank you in advance for your answer,

Best regards

[ASAD]

**Second e-mail survey to Investigators (PIs and/or associate investigators) of studies identified as having no primary publication**

Pr XXX,

I contacted you recently to ask about the existence of publication(s) related to your study.

First, I would like to thank you for your reply. This new request for information will enable me to complete the work I have started as part of my Master placement. This will therefore be my last email, apart - of course - from feedback on the results of our survey, which is planned once the analysis is complete.

I would like to ask you these final questions:

**Results of your study for the primary endpoint:**

☐ Positive

☐ Negative

☐ Not known

**Status of your study:**

☐ No planned primary publication*.

Primary publication pending*:

☐ pre-analysis/analysis

☐ drafting

☐ submission

☐ evaluation

**Reason(s) for absence of a primary publication*:**

☐ Premature study termination

☐ Duplication with a recent publication

☐ Insufficient number of patients included

☐ Analysis results negative/not consistent with the primary endpoint

☐ No consensus on authors' positioning

☐ Lack of time

☐ Lack of available skills (e.g. lack of statistical support)

☐ Delay in obtaining results/blocking factor

☐ Rejection following review(s)

☐ Publication costs too high

☐ 'Other' reason (please specify): ...

* A primary publication focuses on the primary endpoint and presents analyses for all patients included.

Thank you very much, and please do not hesitate to contact me should you require any further information,

[ASAD]
